# Correlation of the temporal and spatial expression patterns of HQT with the biosynthesis and accumulation of chlorogenic acid in *Lonicera japonica* flowers

**DOI:** 10.1038/s41438-019-0154-2

**Published:** 2019-06-01

**Authors:** Yanqun Li, Dexin Kong, Mei Bai, Hanjun He, Haiyang Wang, Hong Wu

**Affiliations:** 1 State Key Laboratory for Conservation and Utilization of Subtropical Agro-Bioresources, South China Agricultural University, Guangzhou, 510642 China; 2 Guangdong Technology Research Center for Traditional Chinese Veterinary Medicine and Natural Medicine, South China Agricultural University, Guangzhou, 510642 China; 3 Guangdong Key Laboratory for Innovative Development and Utilization of Forest Plant Germplasm, South China Agricultural University, Guangzhou, 510642 China

**Keywords:** Non-model organisms, Secondary metabolism

## Abstract

Hydroxycinnamoyl-CoA quinate transferase (HQT) is one of the key enzymes in the biosynthesis of chlorogenic acid (CGA) in the flowers of *Lonicera japonica*. However, the spatiotemporal expression patterns of *HQT* and its relationship to the dynamics of CGA biosynthesis, transport, and storage remain largely unknown. In this study, we collected *L. japonica* flower samples at different growth stages (S1–S6) and examined the spatiotemporal expression pattern of *HQT* and the dynamic accumulation patterns of CGA using a combination of molecular and cytological techniques. Our results suggest that the spatiotemporal expression pattern of *HQT* is directly correlated with dynamic changes in CGA accumulation and distribution in *L. japonica* flowers. We further show that CGA is synthesized primarily in the cytoplasm and chloroplasts. CGA synthesized in the cytoplasm first accumulates in specialized vesicles and is then transferred to large central vacuoles for storage by fusion of CGA-containing vesicles with vacuoles. Furthermore, CGA synthesized in the chloroplasts appears to be transferred into the vacuoles for storage by direct membrane fusion between the tonoplast and the disrupted chloroplast membranes. Collectively, our results suggest that CGA is synthesized in chloroplasts and cytoplasm and finally transferred to the vacuole for long-term storage.

## Introduction

*Lonicera japonica* Thunb., native to eastern Asia, blooms white and yellow flowers with sweet scents in the summer^1^. The dried flower bud is named Flos Lonicerae Japonicae (FLJ), which is commonly used as an antipneumonia, antivirus, antioxidant, and antitumor agent and to enhance liver bile protection^2–6^. FLJ is also widely used as a food additive in beverages and healthy teas^6–8^. Previous studies have shown that FLJ has abundant polyphenols with high antioxidant activities^6,9–13^. Among these polyphenols, chlorogenic acid (CGA) is considered the major bioactive component because of its numerous bioactive functions, such as free-radical scavenging, antioxidation, antivirus, and anticancer activities, and its ability to improve the body’s own defenses^12–16^. Therefore, CGA has been chosen as one of the important standard compounds to evaluate FLJ quality^2,17,18^.

CGA refers to a family of related polyphenol esters formed between hydroxycinnamic acids (caffeic acid, ferulic acid, and *p*-coumaric acid) with quinic acid^19,20^ and is widely distributed in plants of the Caprifoliaceae, Compositae, Solanaceae, and Rubiaceae families^11,21^. The biosynthesis of CGA involves the sequential action of several enzymes, including phenylalanine-ammonia-lyase (PAL), cinnamate 4-hydroxylase (C4H) and 4-coumarate-CoA ligase, generating *p*-coumaroyl-CoA, which serves as the precursor for both phenolic acid and flavonoids^22,23^. Three possible routes of CGA biosynthesis have been proposed. In one route, caffeoyl-CoA and quinic acid are catalyzed by hydroxycinnamoyl-CoA quinate transferase (HQT) to form CGA. In the second route, caffeoyl glucoside is proposed to be the activated intermediate, and the third possible pathway involves the synthesis of *p*-coumaroyl quinate and subsequent hydroxylation of *p*-coumaroyl quinate to form CGA, catalyzed by an acyl transferase and *p*-coumarate 3′-hydroxylase (C3′H), respectively^21^. Regardless of the specific routes utilized to synthesize CGA, the biosynthesis of phenolic compounds is generally believed to be primarily regulated at the transcriptional level^22,24,25^. Previous studies have demonstrated that HQT is a rate-limiting regulatory enzyme in the biosynthetic pathway of CGA in several plant species, including tomato, coffee, and artichoke^21,26–28^. For example, overexpression of *HQT* in tomato results in higher accumulation of CGA, whereas RNAi suppression of *HQT* causes a significant reduction in CGA level^21^. Recently, studies showed that RNAi suppression of *HQT* in potato plants also causes over 90% reduction in CGA levels in potato tubers^24^ and that *HQT* transcript levels correlate with CGA levels in processing potato tubers^25^. Interestingly, another study showed that in potatoes with naturally varying amounts of CGA, HQT enzyme activity but not *HQT* expression levels were well correlated with CGA content^28^. Other studies also reported a better correlation between the transcript levels of hydroxycinnamoyl-CoA shikimate/quinate hydroxycinnamoyl transferase (*HCT*), rather than *HQT*, with CGA in potatoes with varying amounts of CGA owing to development, drought, or other environmental cues^24,29^. Hence, CGA biosynthesis is subject to dynamic regulation by metabolic feedback and environmental factors.

The CGA biosynthesis pathway in *L. japonica* has been well studied. Pu et al.^30^ showed that heterologously expressed LjC3′H protein is effective in converting *p*-coumaroyl quinate to CGA in vitro, whereas Yuan et al.^17^ suggested that *LjC4H2* may be one of the critical genes regulating CGA biosynthesis in *L. japonica*. On the other hand, Peng et al.^31^ showed that HQT can catalyze quinic acid and caffeoyl-CoA to synthesize CGA. Furthermore, the same group detected a positive correlation between CGA content and the expression of *HQT* in *L. japonica* callus transformed with *HQT* transgenes (overexpression or RNAi silencing)^32^. These findings led the authors to conclude that the HQT-mediated pathway represents the major route of CGA biosynthesis in *L. japonica*. Consistent with this finding, an early study showed that the *HQT* transcript level is positively correlated with the content of CGA in different parts of *L. japonica* (buds and stems)^31^. Moreover, we recently reported that the dynamic changes of CGA content throughout the six growth stages of *L. japonica* are directly affected by variations in HQT activity^23^. Despite the progress made in recent years, the tissue- and developmental regulation of *HQT* expression, its enzymatic activity and how it correlates with the dynamic changes in the sites of CGA biosynthesis, accumulation, and storage at different developmental stages of *L. japonica* flowers still have not been explored.

In this study, we utilized a combination of various cytologic techniques, including in situ hybridization, immunofluorescence, and immune-cytochemical localization, to explore the temporal and spatial expression patterns of the *HQT* gene and HQT protein and the sites of biosynthesis and distribution of CGA in *L. japonica* floral organs at different developmental stages. Our results not only reveal a strong correlation between *HQT* expression and CGA biosynthesis but also provide novel insights into the subcellular sites of CGA biosynthesis, as well as its transport and storage.

## Materials and methods

### Plant materials

*L. japonica* plants were grown in the medicinal plant garden at the South China Agricultural University in Guangzhou, Guangdong province, China. All experimental materials were authenticated by Dr. Qiner Yang of South China Botanical Garden, Chinese Academy of Sciences. The experimental materials cultivation, management, and collection were conducted according to a previously described method by Kong et al.^23^.

### High-performance liquid chromatography (HPLC) determination

The ovaries, stamens, and pistils were first removed from the collected flower samples, which were then assessed for CGA content. The CGA content in collected samples was measured according to a previously described method by Kong et al.^23^.

### RNA extraction and analysis

Total RNA was extracted from samples with the E.Z. N.A. Plant RNA Kit (Omega Biotek) according to the manufacturers’ protocols. Five to ten flowers (ovaries, stamens, and pistils removed) at different growth stages (S1–S6) collected from a healthy plant of *L. japonica* were used for total RNA extraction. The RNA concentration was determined using a NanoDrop-2000 (Thermo Scientific, USA). One microgram of total RNA was used to synthesize first-strand cDNA using a reverse transcription kit (Takara, China) in a 20-μL system. Quantitative real-time PCR (qRT-PCR) was performed using the method described by He et al.^33^. The primers for *HQT* were TGAGATCCTAGCTGCCCACT and TGGCTGTGAACACCACATTT. The primers for the *actin* gene were ATGATGCTCCCAGGCAGTTT and ATTGGGCTTCATCACCGACAT, and the relative expression of *HQT* was calculated by the 2^−ΔΔCT^ method^34^. Each value was the mean of three biological replicates.

### Protein extraction and western blot

The flowers of *L. japonica* were collected at growth stages S1–S6, and the ovary, pistil, and stamen were removed. Then, 0.30 g of fresh material was weighed, placed into a liquid nitrogen pre-cooled mortar, supplemented with 10% (w/v) g polyvinylpolypyrrolidone and ground quickly under liquid nitrogen. Next, the powder was transferred to a 10-mL centrifuge tube, 5 mL of 200 mm Tris-HCl (pH 7.5) containing 25% glycerol (v/v), 0.1 m dithiothreitol and 1 mm phenylmethanesulfonyl fluoride (protease inhibitor) were added, and the sample was shaken vigorously with a vortex shaker for 1 min. Afterwards, the extract was centrifuged at 10,000 g for 20 min (4 °C). Then, the protein extract was subjected to western blotting, and the protein contents in different samples were determined by Bradford assay^35^ with some modifications as follows. Standard bovine serum protein (BSA) solution (0.1 mg/mL) was diluted into a series of solutions with different concentrations (10 μg/mL, 20 μg/mL, 40 μg/mL, 60 μg/mL, and 80 μg/mL) with deionized water. The standard curve was plotted with the absorbance versus (*y*) the BSA concentration (*x*, μg/mL) of each sample. The regression equation was *y* = 0.0145 × + 0.0117 with an *R*^2^ value of 0.9997 (Figure S1). The test tubes were numbered, and then 5 ml Coomassie Brilliant Blue G-250 solution (0.01% (w/v) Coomassie Brilliant Blue G-250, 4.7% (w/v) ethanol, and 8.5% (w/v) phosphoric acid were added to each tube either by inversion or vortexing. The absorbance at 595 nm was measured after 10 min. A blank was prepared with equal amounts of deionized water and 5 mL Coomassie Brilliant Blue G-250 solution. The weight of protein was plotted against the corresponding absorbance, resulting in a standard curve used to determine the protein concentrations of the samples.

For preparation of the anti-HQT-specific polyclonal antibodies, the N-terminal fragment (1–46 aa) of the *HQT* coding region was cloned into the pGEX-4T-1 expression vector and expressed in the *Escherichia coli* strain Rosetta (DE3). The target immunogen was purified from inclusion bodies, solubilized in urea and then mixed with Freund’s adjuvant for injection into two Japanese big-ear rabbits for in vivo immunization at the Wuhan AB Clonal Biological Technology Co., Ltd. (injected four times over a 70-day period). Anti-HQT serum was collected, and the reactivity to the antigen was determined by enzyme-linked immunosorbent assay (ELISA). The antiserum was purified using an affinity column conjugated with purified recombinant HQT protein. For immunoassays, the purified protein or an equivalent amount of total protein from *L. japonica* floral organs of various growth stages was separated by electrophoresis on 10% (w/v) sodium dodecyl sulfate polyacrylamide gel electrophoresis and then transferred onto a polyvinylidene difluoride membrane. Afterwards, the membrane was incubated with the purified anti-HQT antibodies (TBST dilution 1:1000) overnight at 4 °C, followed by color development with horseradish peroxidase-enhanced chemiluminescence assay.

### Frozen sections

The corolla tube of *L. japonica* was cut according to the method of Li et al.^36^. Each section was subjected to Neu’s reagent (1,2-aminoethyl diphenylborinate (Sigma-Aldrich), anhydrous methanol solution). After 30 s, the section was sealed with 10% glycerin solution and then examined under a fluorescence microscope (Leica DMLB) (fluorescent excitation wavelength: 365 nm)^37^.

### Semithin sections

The tissue of the corolla tube of *L. japonica* was cut into pieces with a length of ~ 0.5 mm. The specimens were treated according to the method of Li et al.^38^. Specimens were sectioned on a microtome (Leica RM2155) (1-μm thickness), stained with 0.2% TBO and photographed using a Leica EM UC6 microscope (Leica, Germany).

### In situ hybridization

In situ hybridization was conducted according to the protocols of Jackson^39^ and Bortiri^40^ with some modifications. *L. japonica* corolla tubes at different growth stages were fixed in 4% paraformaldehyde for 16–22 h at 4 °C. For the preparation of the probe, the primers *HQT*_F, 5′- ATGGGAAGTGAAGGAAGTGT-3′ and *HQT*_R, 5′-ATCTCAACTCTTCCTTCATC-3′ were used to amplify a cDNA fragment from the *L. japonica* corolla tube, and the amplification product was cloned into pGEM-T Easy (Roche) and linearized by digestion with *Hin*dIII and *Eco*RI.

### Immunofluorescence localization of HQT

The corolla tube of *L. japonica* was cut into pieces of ~ 1 mm in length. The specimens were fixed in 4% paraformaldehyde for 16–22 h at 4 °C. The paraffin sections were prepared using the method of He et al.^33^. Immunofluorescence signals of HQT protein were determined according to the instructions of the immunofluorescence kit (Boster Biotechnology Co., Ltd., Wuhan, China). The primary antibodies were diluted with phosphate-buffered saline (PBS; 1:100) and incubated at 37 °C for 1 h.

### Immunocytochemistry localization of HQT protein

Immunolabeling was carried out using the method of Fang et al.^41^ with some modifications. First, 2-mm-long sections in the center of *L. japonica* corolla tubes at different growth stages were fixed in a solution (0.5% glutaraldehyde (v/v), 4% paraformaldehyde (w/v), 100 mm PBS, pH 7.2) for 12 h at 4 °C. Ultrathin sections (60–70 nm) were prepared using a Leica EM UC6 ultramicrotome (Leica) and collected by Formvar-coated nickel grids (Gilder Grids). The primary anti-HQT antibody was used at a 1:10 dilution, and the goat anti-rabbit IgG antibody conjugated to 10-nm gold particles (Sigma-Aldrich) was used at a 1:50 dilution. The primary antibodies were substituted with preimmune serum as the control.

### Cytochemical localization of CGA

Colloidal gold with a particle diameter of 15 nm was prepared using the method of Gorshkova et al.^42^. The specimens were prepared as described by Fang et al.^41^. Labeling with the gold-complexed laccase (localization of CGA) in *L. japonica* corolla tube sections was performed according to the method of Gorshkova et al.^42^.

## Results

### Anatomic features of the floral organs of *L. japonica* at different developmental stages

**Fig. 1:**
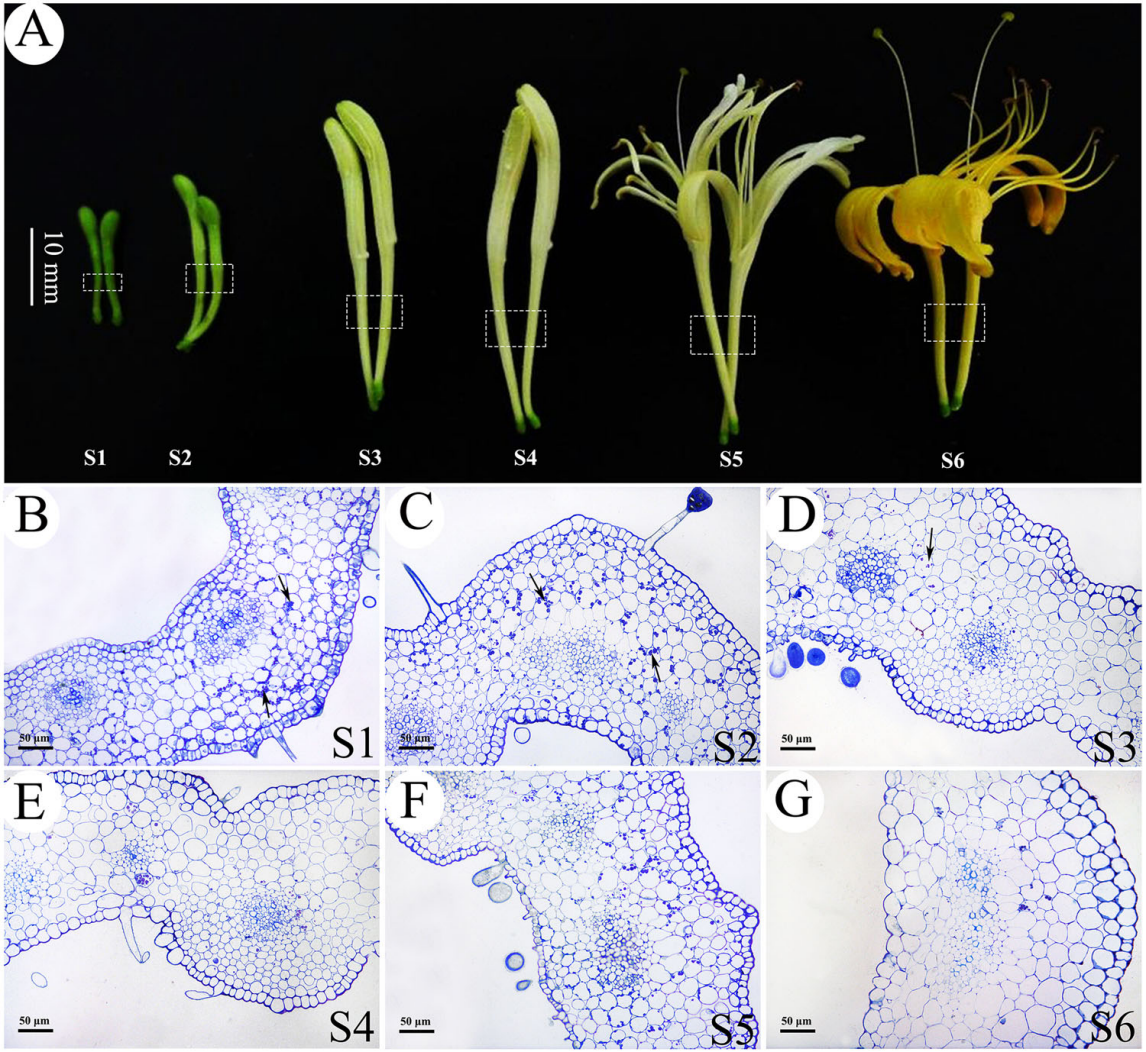
A morphological characteristics of *L. japonica* corolla tubes at different developmental stages. **a** Representative images of *L. japonica* corolla tubes at different developmental stages (S1–S6). The dotted box indicates the segment used for anatomical examination. **b**–**g** Cross-sections of *L. japonica* corolla tubes of the six developmental stages. S1: young alabastrum, S2: green alabastrum, S3: slightly white alabastrum, S4: whole white alabastrum, S5: silvery flower, and S6: golden flower

The main developmental processes of *L. japonica* flowers were divided into six stages as previously described^23^. As shown in Fig. 1a, the external morphology and colors of *L. japonica* flowers were significantly different at the six developmental growth stages. Cellular examination revealed that the tube corolla of *L. japonica* is composed of the inner and outer epidermis, basic tissue and vascular bundle. The inner and outer epidermal cells were tightly arranged, with glandular and nonglandular trichomes (Fig. 1b–f). The basic parenchyma cells were loosely arranged, with a large central vacuole (Fig. 1b–g). The parenchyma cells contained numerous starch grains during the S1–S3 stages (Fig. 1b–d).

### Dynamic changes in *HQT* expression, HQT protein accumulation and CGA content levels

**Fig. 2:**
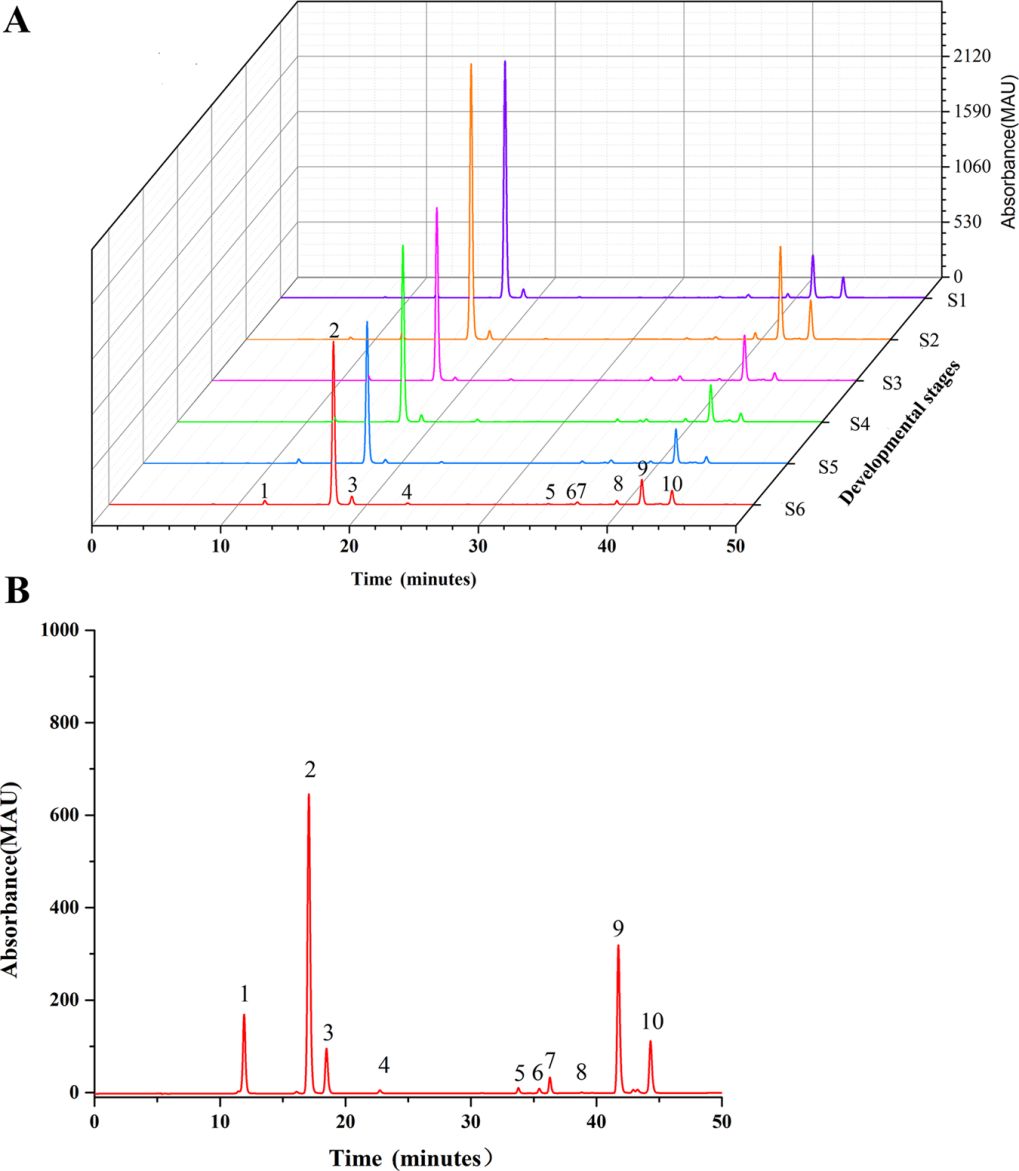
HPLC chromatograms of the extracts from *L. japonica* flowers at different developmental stages. **a** Chromatograms of extracts from *L. japonica* flowers (with ovaries, stamens and pistils removed) at stages S1–S6. **b** Chromatograms of seven standard phenolic acids and three standard flavonoids injected in a mixture. Peaks 1, 2, 3, 4, 5, 6, 7, 8, 9, and 10 represent 5-caffeoylquinic acid, chlorogenic acid, 4-caffeoylquinic acid, caffeic acid, rutin, hyperoside, luteoloside, 3,4-dio-caffeoylquinic acid, 3,5-dio-caffeoylquinic acid, and 4,5-dio-caffeoylquinic acid, respectively

**Fig. 3:**
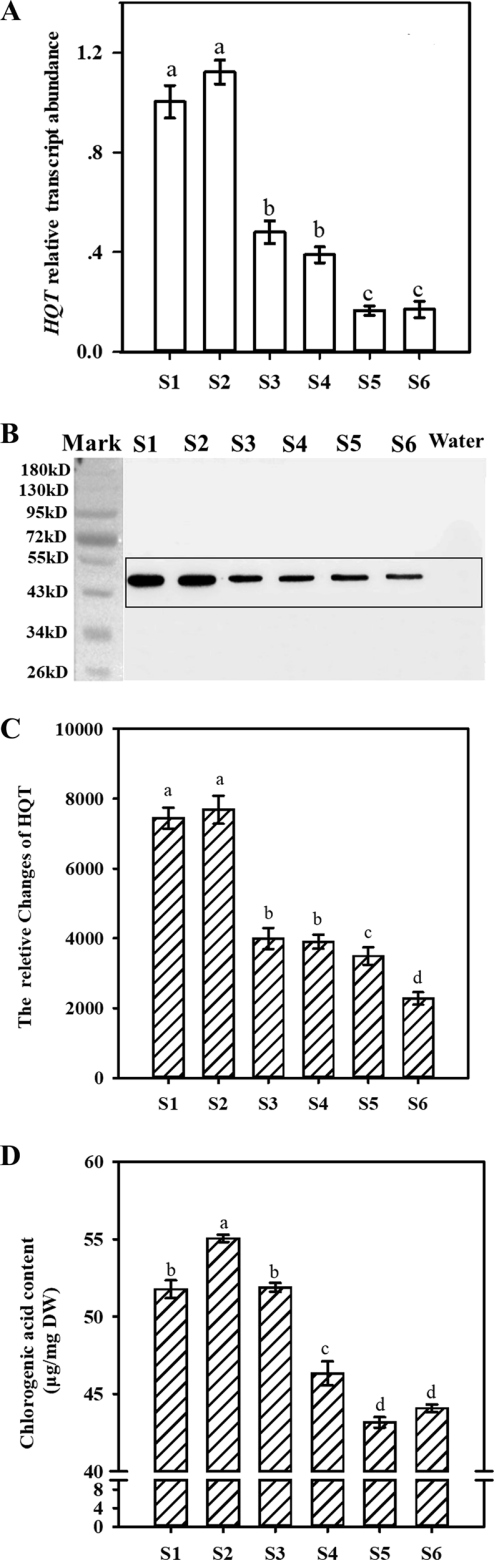
Measurement of *HQT* expression levels, protein accumulation, and CGA contents in *L. japonica* flowers at different developmental stages. **a** Relative expression levels of *HQT* in *L. japonica* flowers at different developmental stages. **b** Western blot assay showing variations in HQT protein accumulation in *L. japonica* flowers at different growth stages. **c** Quantification of HQT protein levels in *L. japonica* flowers at different growth stages. **d** Quantification of CGA levels in *L. japonica* flowers at different growth stages

The flower of *L. japonica* is mainly composed of the corolla tube, stamens and pistils. To ensure consistent results, we chose to examine the corolla tube, the organ with the most abundant CGA^23^, in the present study. To investigate the relationships between the expression patterns of *HQT* and the dynamic accumulation of CGA in *L. japonica* corolla tubes at different growth stages (S1–S6), we first measured the dynamics of *HQT* transcript expression in *L. japonica* corolla tubes at the six growth stages using qRT-PCR. The relative expression of *HQT* was higher at the S1–S2 stages than at the later four stages, with the lowest expression level detected at the S5–S6 stages (Fig. 3a). To detect the dynamics of HQT protein accumulation, we expressed the N-terminal domain (1–144 aa) of the HQT protein in *E. coli*, and recombinant protein was purified to immunize rabbits (Figure S2). The antiserum was collected and affinity purified with immobilized recombinant protein. ELISA was performed to determine the reactivity and specificity of the anti-HQT polyclonal antibodies (Figure S3, S4). We then used purified anti-HQT polyclonal antibodies to perform western blot analysis with *L. japonica* flower samples at six growth stages (S1–S6). A single band of ~ 48.3 kD was detected, indicating the specificity of the antibody (Fig. 3b). Then, using western blot, we showed that the accumulation of HQT protein exhibited a sequential decreasing trend in the corolla tubes during flower development, being highest at the S1–S2 stages and lowest at the S6 stage (Fig. 3b, c). This pattern suggests that the protein level of HQT correlates well with the expression dynamics of the *HQT* transcript level. In addition, measurement of CGA contents in the corolla tubes at the six growth stages using HPLC showed that the levels of CGA content were higher at the S1–S3 stages (51.76–55.05 μg/mg) than at the S4–S6 stages (43.16–46.07 μg/mg) (Figs. 2 and 3d). Thus, CGA content levels also correlate well with the changes in the levels of *HQT* mRNA and its encoded protein.

### Association of the tissue-specific expression of *HQT* and its encoded protein with the distribution of CGA in *L. japonica* corolla tubes at different growth stages

**Fig. 4:**
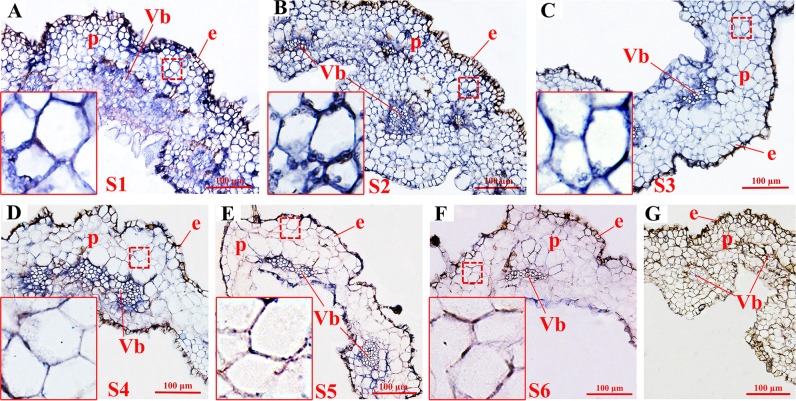
Analysis of *HQT* expression by in situ hybridization in the *L. japonica* corolla tube at stages S1–S6 (blue signal). **a–b** Abundant signals of *HQT* were detected in the whole corolla tube, including the inner and outer epidermal cells and parenchyma cells, especially in the vascular parenchyma cells at stages S1–S2. **c**–**d** The *HQT* signals were significantly reduced in the coronal parenchyma cells but remained strong in the vascular parenchyma cells at stages S3–S4 compared with that at stages S1–S2. **e–f** The *HQT* signals further decreased, with weak blue signals detected only in the epidermal and vascular bundle cells throughout the corolla tubes at the S5 and S6 stages. Insets in **a**–**f** show enlarged views of the corresponding images. **g** An *HQT* mRNA sense probe was used as the negative control for in situ hybridization. Bar = 100 μm for all images (excluding the insets). **a** Young alabastrum (S1); **b** green alabastrum (S2); **c** slightly white alabastrum (S3); **d** whole white alabastrum (S4); **e** silvery flower (S5); **f** golden flower (S6). e: epidermis; p: parenchymal cell; Vb: vascular bundle

**Fig. 5:**
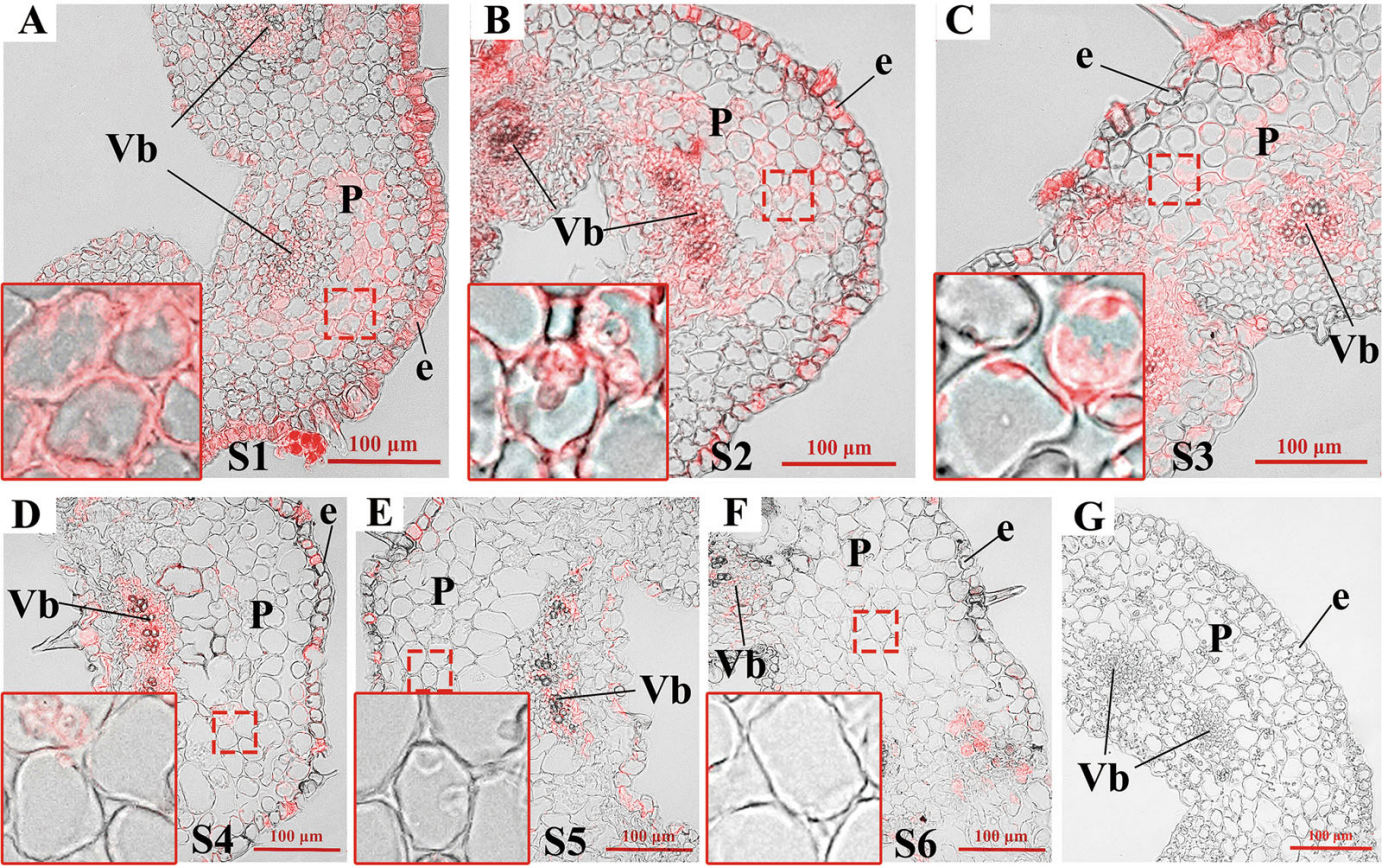
Analysis of HQT protein accumulation by immunofluorescence localization in the *L. japonica* corolla tube at stages S1–S6. **a**–**b** Abundant HQT proteins (red fluorescence signals) were detected in the inner and outer epidermal cells and parenchyma cells, especially in the vascular parenchyma cells at the early growth stages (S1–S2). **c**–**d** The red fluorescence signals were significantly reduced in the coronal parenchyma cells but remained strong in the vascular parenchyma cells compared with those at stages S1–S2. **e**–**f** Weak red fluorescence signals were observed in the epidermal and vascular bundle cells for the whole corolla tubes at stages S5–S6. Insets in **a**–**f** show enlarged views of the corresponding images. HQT protein signals were observed in the cytoplasm, and these red signals in the parenchyma cells of corolla tubes were less intense in the S4–S6 stages than in the earlier stages (S1–S3). **g** Negative controls of immunofluorescence localization. Preimmune serum was used to substitute for the anti-HQT antibodies as the negative control for localization of HQT at the S2 growth stages. Bar = 100 μm for all images (excluding the insets). **a** young alabastrum (S1); **b** green alabastrum (S2); **c** slightly white alabastrum (S3); **d** whole white alabastrum (S4); **e** silvery flower (S5); **f** golden flower (S6). e: epidermis; p: parenchymal cell; Vb: vascular bundle

**Fig. 6:**
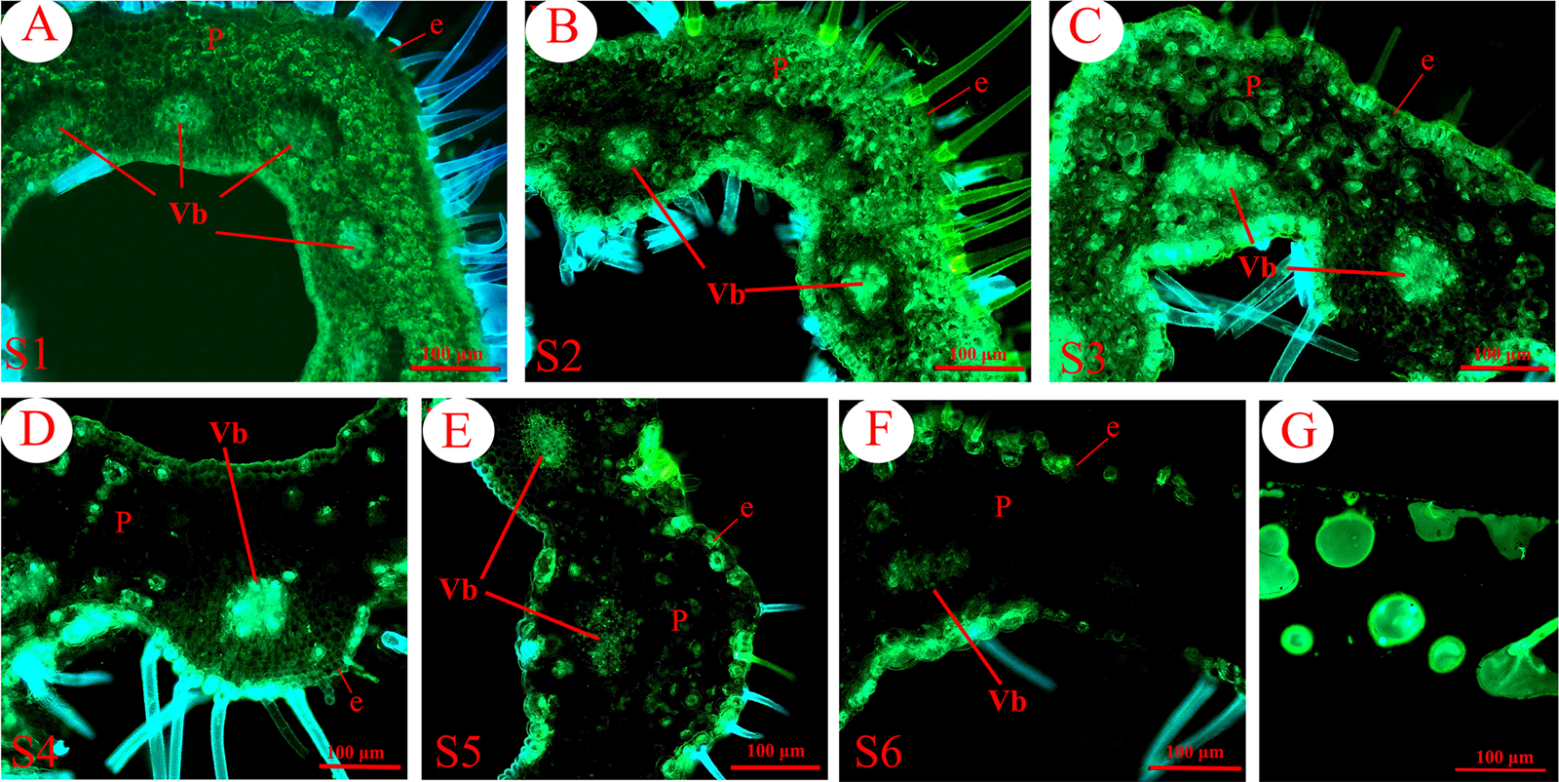
Distribution pattern of CGA in the *L. japonica* corolla tube at stages S1–S6. **a**–**c** Green fluorescence shows phenolic compounds with Neu treatment (indicated by arrow), which were detected near the inner and outer epidermal cells, parenchyma cells, and vascular bundles. **d** CGA in parenchyma cells was significantly reduced compared to that in the S1–S3 stages. Strong fluorescence signals were retained only in the outer and inner epidermal cells and the vascular bundles at stage S4. **e**–**f** Further reductions in CGA signals were observed in the parenchyma cells, the vascular bundles and inner and outer epidermal cells at stages S5–S6. **g** Fluorescence image of the CGA standard. **a** young alabastrum (S1), **b** green alabastrum (S2); **c** slightly white alabastrum (S3); **d** whole white alabastrum (S4); **e** silvery flower (S5); **f** golden flower (S6); Bar = 100 μm for all images. e: epidermis; p: parenchymal cell; Vb: vascular bundle

To further investigate the relationship between *HQT* expression and CGA biosynthesis, we employed in situ hybridization to determine the expression pattern of *HQT* (Fig. 4) and immunofluorescence to determine the distribution pattern of its encoded protein in the corolla tubes of *L. japonica* at different growth stages (Fig. 5). Meanwhile, the sites of CGA accumulation in the corolla tube cells were detected throughout the six growth stages (S1–S6) using a fluorescent histochemical localization assay (Fig. 6). At stages S1 and S2, abundant signals of *HQT* (Fig. 4a, b, blue signals) and its encoded HQT protein (Fig. 5a, b, red fluorescence signals) were detected throughout the corolla tubes, including the inner and outer epidermal cells and parenchyma cells, especially in the vascular parenchyma cells. Further comparisons of the enlarged images (insets) revealed that the blue and red signals were distributed primarily in the cytoplasm (Figs. 4a and 5a) and chloroplasts (Figs. 4b and 5b) at the early stages of corolla tube development (S1–S2). At the S3 stage, the central vacuoles of corolla tube cells increased markedly in volume, and the cytoplasm was squeezed to the edges of cells. The signals of *HQT* mRNA and its encoded protein became weaker in the coronal parenchyma cells but remained strong in the vascular parenchyma cells (Figs. 4c and 5c). Similar to the expression pattern of *HQT* and HQT protein accumulation, CGA accumulation (green signal, Fig. 6g) was detected near the inner and outer epidermal cells, parenchyma cells, and vascular bundles of the *L. japonica* corolla tubes at stages S1–S3 (Fig. 6a–c). By stage S4, the *HQT* mRNA (blue signal, Fig. 4d) and HQT protein (red signal, Fig. 5d) signals in the parenchyma cells of corolla tubes were significantly reduced compared with their levels in stages S1–S3, with only a weak signal still present; however, strong signals were maintained in the vascular bundles and the outer and inner epidermal cells. Consistent with the dynamic changes in *HQT* mRNA and HQT protein expression, the accumulation of CGA was also significantly reduced in the parenchyma cells but remained strong in the outer and inner epidermal cells and the vascular bundles at the S4 stage (Fig. 6d). At the S5 and S6 stages, the levels of *HQT* transcript and its encoded protein further decreased, with weak blue signals (Fig. 4e, f) and red signals (Fig. 5e, f) being detected only in the epidermal and vascular bundle cells of the corolla tubes. Similarly, the CGA signals were very weak in the parenchyma cells, with some residual, scattered green fluorescence signals still detected in the vascular bundles and the inner and outer epidermal cells (Fig. 6e, f). These observations suggest that the temporal and spatial accumulation of CGA is well correlated with the expression pattern of *HQT* and its encoded protein.

### Synthesis and storage sites of CGA

**Fig. 7:**
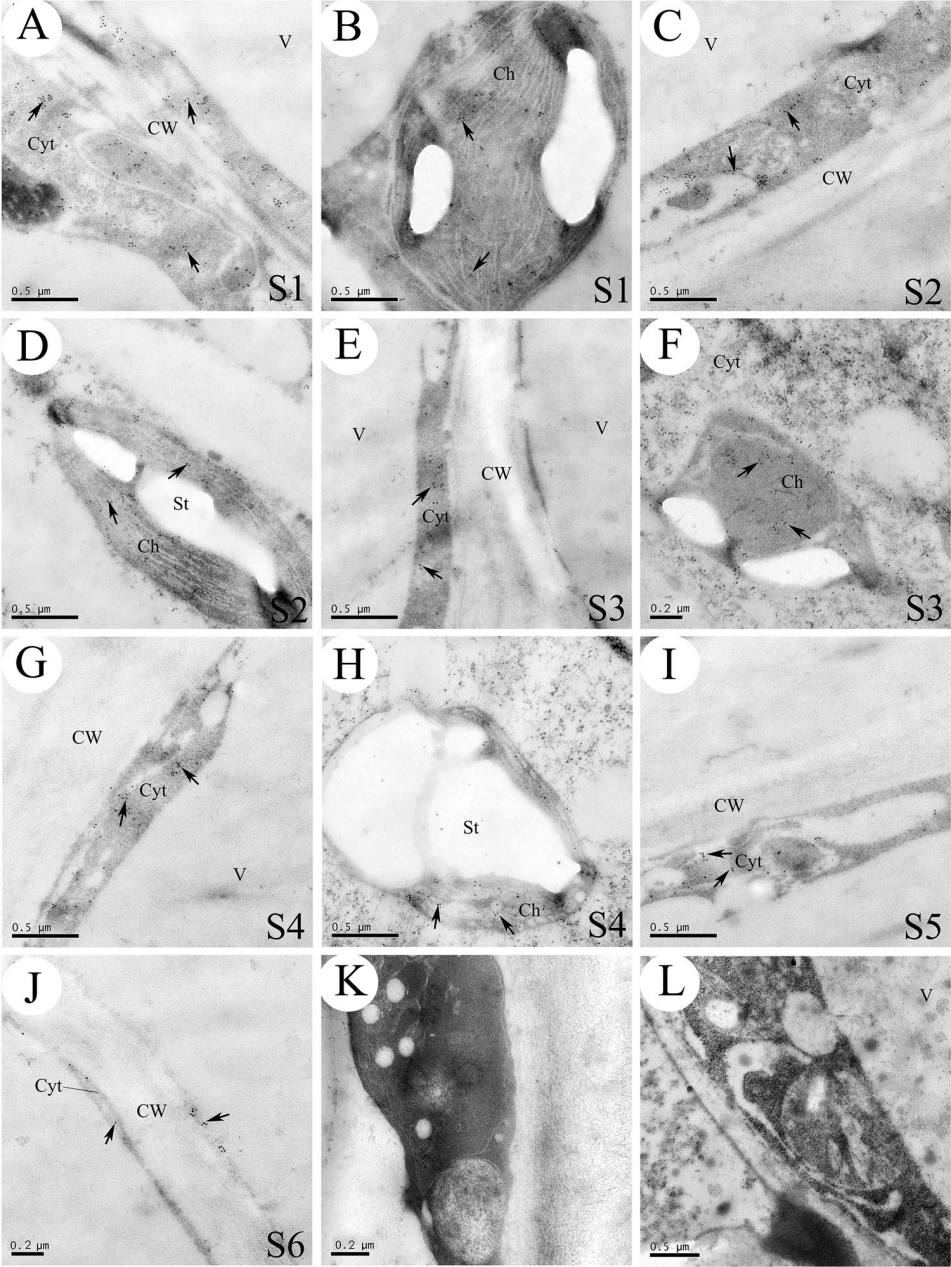
Immuno-cytochemical localization of the HQT protein in *L. japonica*. **a**–**d** Abundant anti-HQT-IgG particles were detected in the cytoplasm **a**, **c** and chloroplasts **b**, **d**. No particles were detected in the cell walls at stage S1–S2. **e**–**h** The cytoplasm was squeezed into a thin layer at the edge of the cell **e**. Many anti-HQT-lgG particles were detected in the cytoplasm **e**, **f**, **g**, and the numbers of anti-HQT-lgG particles were reduced in chloroplasts accompanied by an increase in starch grain size **h**. **i**–**j** The cytoplasm became quite thin, and only a few scattered anti-HQT-lgG particles were detected in the cytoplasm at the S5–S6 stages; no anti-HQT-lgG particles were found in the cell wall or vacuole throughout the six growth stages (S1–S6). **k** Control corolla tube cell section incubated with preimmune serum. **l** A control section that was only incubated with preimmune serum. Black arrows indicate the anti-HQT particles; CW: cell wall; V: vacuole; Ve: vesicle; St: starch grain; Cyt: cytoplasm

To further validate the critical role of the HQT enzyme in the biosynthesis of CGA in the corolla tube of *L. japonica*, we first used anti-HQT-immunogold particles to investigate the subcellular localization of the HQT protein in flowers of *L. japonica* at different growth stages. At the early stages (S1 and S2), the cytoplasm of parenchyma cells was dense (Fig. 7a, c), and the chloroplasts had a normal ultrastructural organization, with a typical arrangement of stroma and grana thylakoids. Most chloroplasts contained starch grains (Fig. 7b, d). At this stage, abundant anti-HQT-immunogold particles were detected in the cytoplasm near the vacuole (Fig. 7a, c) and in the chloroplasts (Fig. 7b, d). The dual localization of the HQT protein is consistent with the prediction of a putative plastid-targeting signal using the TargetP and ChloroP prediction tools (http://www.cbs.dtu.dk/services/TargetP/ and http://www.cbs.dtu.dk/services/ChloroP/, Table S1)^43^ and cytoplasm targeting using the Cell-PLoc package (http://chou.med.harvard.edu/bioinf/Cell-PLoc)^44^. At the middle stages (S3 and S4), we observed numerous small vesicles coalesced with each other to form larger vacuoles, and the cytoplasm was squeezed into a thin layer surrounding the edges of the cell (Fig. 7e, g). The chloroplast grana were completely ruptured and disappeared at these stages, and the anti-HQT-immunogold particles were mainly located in the cytoplasm (Fig. 7e, g) and chloroplasts (Fig. 7f, h). At the last two stages (S5 and S6), the large central vacuole occupied most of the cell, and the cytoplasm became thin, with only a few scattered anti-HQT-immunogold particles observed in the cytoplasm (Fig. 7i, j).

Next, we used a cytochemical technique to examine the synthesis sites of CGA in the corolla tube of *L. japonica*. Laccase is a polyphenol oxidase that catalyzes the oxidization of substrates such as polyphenols and aromatic polyamines and forms laccase-gold complexes with colloidal gold particles. Thus, the distribution and storage sites of the phenolic compounds were determined by examining the distribution of gold particles under a transmission electron microscope^45^. Consistent with an earlier report^46^, we found that CGA is a major polyphenolic compound among the phenolic acid compounds (seven phenolic acids and three flavonoids) present in the *L. japonica* flowers at different growth stages (up to 72.3% of the total polyphenols, Fig. 2a). Therefore, the distribution sites of laccase-gold particles in the tube corolla cells of *L. japonica* served as a good indicator of the subcellular localization of polyphenols and CGA in the cells.

**Fig. 8:**
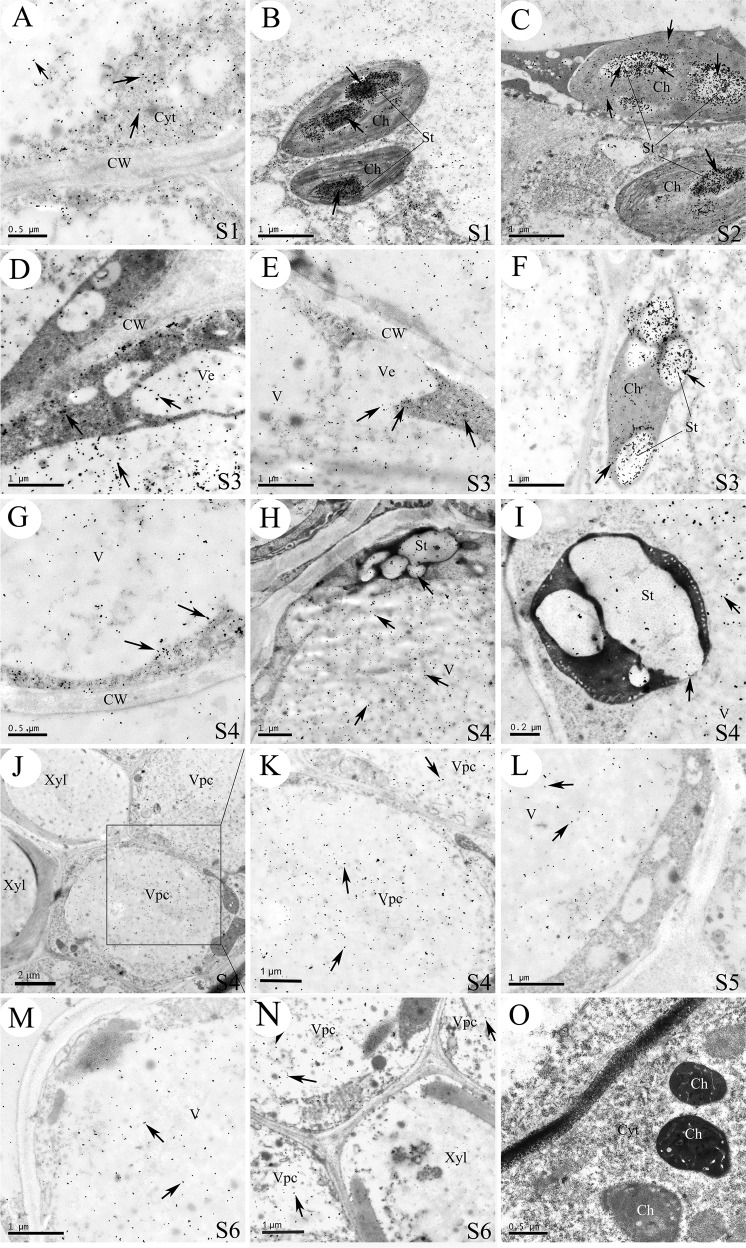
Cytochemical localization of the phenolic compounds in *L. japonica* corolla tube cells at stages S1–S6. **a–c** At the growth stages of S1–S2, the distribution of some laccase-gold particles in the cytoplasm **a** and chloroplasts **b**, **c**. Clustered laccase-gold particles were detected on the chloroplast starch grains **b**, **c**. **d**–**f** At the S3 stage, coalescence of vesicle and the large central vacuole resulted in the transfer of more laccase-gold particles from the small vacuoles to the large central vacuole **d**, **e**. In addition, a large number of laccase-gold particles in starch grains began to transfer to the cytoplasm **f**. **g**–**k** At the S4 stage, laccase-gold particles were transferred from the cytoplasm to vacuole **g**. Starch grains in the chloroplasts gradually began to degrade **h**, and the gold particles in the starch grains were transferred to the cytoplasm or vacuoles by fusion of the plastid membranes with the vacuole membranes **i**, resulting in an increase in laccase-gold particles in vacuoles. Note that at this stage, many laccase-gold particles were scattered in the parenchymal cells of vascular bundles **j**, **k**. **l**–**n** At the S5–S6 stages, laccase-gold particles were mainly stored in the large central vacuole, with little or no labeling detected in the cytoplasm and on the cell wall **l**, **m**. Some laccase-gold particles were detected in the parenchymal cells of the vascular bundles **n**. **o** Colloidal gold solution without laccase was used as a control, and no colloidal gold particles were detected in the cell. Black arrows indicate laccase-gold particles. Ch: chloroplast; CW: cell wall; Xyl: xylem; Vpc: vascular bundle parenchyma cells; V: vacuole; Ve: vesicle; St: starch grain; Cyt: cytoplasm

Using this method, we found that at the early stages (S1 and S2), abundant laccase-gold particles were detected in the cytoplasm (Fig. 8a) and chloroplasts of parenchyma cells (Fig. 8b, c), similar to the labeling of anti-HQT-immunogold particles. Notably, we found that many laccase-gold particles clustered on the starch granules in the chloroplasts (Fig. 8b, c). Scattered laccase-gold particles were also observed in the vacuoles, and some vesicles with scattered gold particles were merging into a large vacuole (Fig. 8a–c). At the middle stages (S3 and S4), small vesicles coalesced with each other to form a larger vacuole, and the cytoplasm was squeezed into a thin layer surrounding the edge of the cell (Fig. 8d, e). The grana of chloroplasts completely disappeared, and the starch grains were larger at these stages than at the earlier stages (Fig. 8f). More laccase-gold particles were observed in the large central vacuole, probably owing to membrane fusion of small vesicles containing laccase-gold particles (Fig. 8d, e). Generally, numerous laccase-gold particles were scattered or gathered in the vascular parenchyma cells (Fig. 8j, k), and the vacuoles were strongly labeled with laccase-gold particles (Fig. 8k). At these stages, starch grains in the chloroplasts gradually began to degrade, and the laccase-gold particles on the starch grains in the chloroplasts were released to the cytoplasm or vacuoles by fusion of the plastid envelope with the tonoplast (Fig. 8h, i), resulting in an increase in laccase-gold levels in the vacuoles and decreased laccase-gold levels on the starch grains (Fig. 8h, i). At the last two stages (S5 and S6), the large central vacuole occupied most of the cell. Laccase-gold particles were still gathered in the vacuoles of the parenchyma cells (Fig. 8l–n), but the signals were notably weaker at these stages than at the early and middle stages (S1–S4). Few or no laccase-gold particles were observed in the cytoplasm at the S5 and S6 stages (Fig. 8l, m).

Notably, no anti-HQT-immunogold particles were observed on the cell wall or inside the vacuoles throughout the six growth stages (Fig. 7). In addition, no scattered anti-HQT-immunogold particles were observed in the control, in which the HQT antibodies were replaced by the preimmunization serum (Fig. 7k) or HQT antibodies were omitted (Fig. 7l). Similarly, no gold labeling by colloidal gold solution without laccase was observed in the control (Fig. 8o).

Together, the above results indicated that CGA was primarily synthetized in the chloroplasts and cytoplasm at the early stages. Only a small amount of CGA was synthetized in the cytoplasm at the late stages. Eventually, CGA was transported to the vacuoles for long-term storage.

## Discussion

### Transcriptional regulation of *HQT* has a predominant role in regulating CGA synthesis in *L. japonica*

Previous studies have accumulated evidence to suggest that the HQT-mediated pathway represents the major route of CGA biosynthesis in *L. japonica*^31,32^. Consistent with this notion, our earlier studies showed that activity is well correlated with dynamic changes in CGA content throughout the developmental stages of *L. japonica*^23^. In this study, we used in situ hybridization, immunofluorescence localization, and fluorescence histochemistry to further study the dynamic correlation between the transcript level of *HQT* and the accumulation sites of its encoded protein with CGA biosynthesis and distribution. These techniques allowed us to systematically examine the expression pattern of *HQT*, its protein accumulation and sites of CGA biosynthesis and storage at the cellular and subcellular levels. We found that *HQT* is expressed at higher levels at the early stages (S1–S2), with the lowest expression detected at the S5–S6 stages (Fig. 3a). Consistent with this observation, western blot analysis revealed that HQT protein accumulation was highest at the S1–S2 stages and gradually decreased, reaching the lowest point at the S6 stage (Fig. 3b, c). Measurement of CGA content showed that the corolla tubes of *L. japonica* had higher CGA expression at the S1–S2 stages than at the S4–S6 stages (Figs. 2 and 3d). Together, these results clearly indicate that expression of *HQT* directly regulates the biosynthesis of CGA, and thus transcriptional regulation of *HQT* expression has a predominant role in the tissue and developmental-stage regulation of CGA biosynthesis in *L. japonica*. Notably, the changes in the expression levels of *HQT* and CGA were not completely consistent at S1–S3 (Fig. 3a–d). The underlying reasons for this discrepancy are not known. We speculate that the reduced expression of *HQT* at the S3 stage might be owing to metabolic feedback inhibition by high levels of CGA accumulated at the S2 stage.

Our detailed cytological analyses for the first time revealed the subcellular expression of *HQT* and accumulation of its encoded protein and CGA. We found that at the early stages (S1 and S2), *HQT* expression and accumulation of its encoded protein and CGA were distributed throughout the corolla tube, including the inner and outer epidermal cells and parenchyma cells, especially in the vascular parenchyma cells (Figs. 4a–c, 5a–c and 6a–c). During the maturation of the floral organs (S4–S6 stages), *HQT* expression, its encoded protein and CGA content were significantly reduced first in the parenchyma cells and epidermal cells and then in the vascular bundles (Figs. 4d–f, 5d–f and 6d–f). Thus, the dynamic changes in the transcript levels of *HQT* and accumulation of its encoded protein correlated well with the dynamic changes in the accumulation of CGA at the six growth stages of *L. japonica*. These results provide additional evidence to support the notion that the *HQT* gene and its encoded protein directly regulate the synthesis and accumulation of CGA throughout developmental growth stages in flowers of *L. japonica*.

As both CGA and lignin are metabolites of phenylalanine and their metabolic pathways share common intermediates and enzymes^47,48^, we speculate that *HQT*, its encoded HQT protein and CGA accumulated near the vascular bundles may provide the necessary precursor or substrate for lignin synthesis at the late stages of flower development in *L. japonica*. Further studies are required to verify this possibility.

Earlier studies have also documented roles of several P450 enzymes, including *LjC3*′*H* and *LjC4H2*, in regulating CGA synthesis in *L. japonica*^17,30^. Interestingly, Qi et al.^46^ reported that *LjPAL1, LjC4H3, LjC3*′*H1*, and *LjHQT* display a similar expression pattern (first decreasing and then increasing), whereas only *LjC3*′*H2* exhibits a similar pattern with CGA contents in buds and flowers of *L. japonica* at different developmental stages. The reasons for the inconsistency regarding the expression pattern of *HQT* with CGA content with our results are not yet clear and remain to be clarified in future studies. Nevertheless, the co-expression pattern of *LjPAL1, LjC4H3, LjC3*′*H1*, and *LjHQT* raises an intriguing possibility that these four genes might be regulated by the same transcription factor(s) as they have similar cis elements in their promoters^49^, which will also be an interesting avenue for future research.

### CGA is synthesized in chloroplasts and cytoplasm and finally transported to vacuoles for long-term storage

Previously, CGA was speculated to be mainly synthesized in chloroplasts and cytoplasm and eventually stored in vacuoles, based on the distribution of CGA in the young and old leaves of coffee^50^. However, cytological evidence supporting such a speculation has remained forthcoming. In this study, we used laccase-labeled gold particles to directly track the distribution and dynamic changes of polyphenols (primarily CGA) in the six developmental stages of *L. japonica* flowers. In addition, we conducted parallel immunolocalization studies of HQT protein and CGA. Our results showed that both HQT protein (detected by anti-HQT- immunogold particle) and CGA (detected by laccase-labeled gold particle) were distributed in both the cytoplasm and chloroplasts during stages S1–S2 (Figs. 7a–d and 8a–c). However, with the development of the corolla tubes, the distribution pattern of the HQT protein became somewhat different from that of laccase-labeled gold particles (presumably CGA). For example, HQT was still mainly found in the cytoplasm at the S5–S6 stages (Fig. 7i, j), whereas only a few or no laccase-labeled gold particles were detected in the cytoplasm. Instead, laccase-labeled gold particles were primarily detected in the central vacuoles (Fig. 8l, m). These observations suggest that CGA is synthesized in the cytoplasm and chloroplasts and later transported to vacuoles for long-term storage, as no HQT was observed in the vacuoles throughout the developmental stages.

Notably, abundant laccase-gold particles were detected around the starch grains at the early growth stages of *L. japonica* corolla tubes (Fig. 8b, c). The signal became significantly weaker as the flower matured, accompanied by degradation of starch granules and rupture of the chloroplasts. These observations led us to speculate that the degradation product of starch grains in the chloroplasts may provide an important precursor for CGA synthesis and that the reduced abundance of laccase-gold particles around the starch grains later might be explained (at least partially) by transfer of CGA into vacuoles for storage at the later stages. Consistent with this notion, previous studies have shown that phenolic acids in *Polypodium vulgare* L. rhizomes were mainly distributed around the amyloplast starch grains^51^ and that starch in the chloroplasts was involved in the synthesis of tannins or phenolic acids^52–54^. In addition, our cellular evidence also suggests that CGA synthesized in the cytoplasm migrated into the large central vacuoles mainly through the aggregation of vesicles and then fusion of the vesicular membranes with the vacuolar membranes. Eventually, CGA was stored in the large vacuoles (Fig. 8d, e). On the other hand, the chloroplast-synthesized CGA appears to be transported to the vacuoles through a different process. At the early stages of *L. japonica* development, the plastid structure was intact, and many laccase-gold particles were enriched inside and around the starch granules (Fig. 8b, c). The starch in the chloroplasts degraded gradually, and the plastid membranes ruptured during floral organ development. The laccase-gold particles in the chloroplasts near the vacuoles appeared to be released directly into the vacuoles by fusion of the plastid membranes with the tonoplast for storage (Fig. 8h, i).

## Supplementary information

Supplemental Material
