# Supplementary material for: Correlation of the temporal and spatial expression patterns of HQT with the biosynthesis and accumulation of chlorogenic acid in Lonicera japonica flowers
Source: Hortic Res. 2019 Jun 1;6:73. doi: 10.1038/s41438-019-0154-2 (PMC6544646; doi:10.1038/s41438-019-0154-2)
Supplement: Supplementary file 1 — Supplemental Material [file 41438_2019_154_MOESM1_ESM.docx]

**Supplemental Information for**

**Correlation of the temporal and spatial expression patterns of HQT with the biosynthesis and accumulation of chlorogenic acid in *Lonicera japonica* flowers**

Yanqun Li^1,2,3,&^, Dexin Kong^1,&^, Mei Bai^1^, Hanjun He^1^, Haiyang Wang^1*^, Hong Wu^1,2,3*^

^1^ State Key Laboratory for Conservation and Utilization of Subtropical Agro-Bioresources, South China Agricultural University, Guangzhou 510642, China

^2^ Guangdong Technology Research Center for Traditional Chinese Veterinary Medicine and Natural Medicine, South China Agricultural University, Guangzhou 510642, China

^3^ Guangdong Key Laboratory for Innovative Development and Utilization of Forest Plant Germplasm, South China Agricultural University, Guangzhou 510642, China

**^&^** These authors contributed equally to this work.

∗Corresponding author: E-mail address: whyang@[scau.edu.cn](mailto:scau.edu.cn), [wh@scau.edu.cn](mailto:wh@scau.edu.cn).

**Figure S1. A Bradford protein assay standard curve produced using BSA at triplicate points of 0, 10, 20, 40, 60, and 80 µg. The data are fit with a linear regression by the line y =0.0145x + 0.0117 with an R^2^ value of 0.9997.**

**
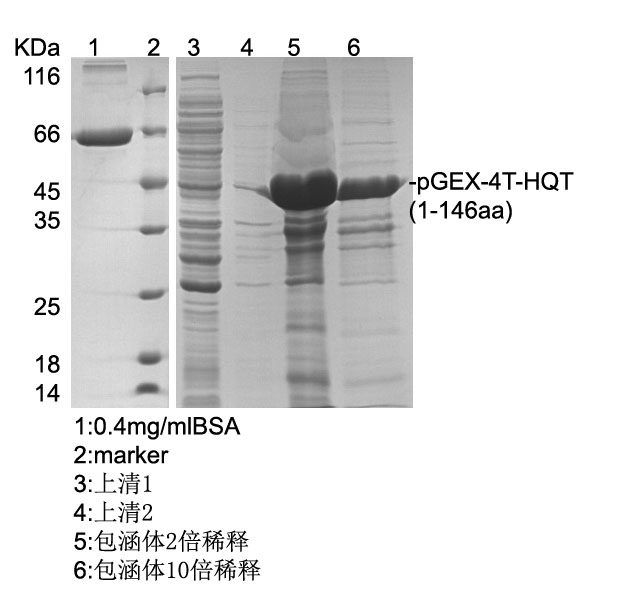
**

**Figure. S2: Expression and purification of HQT recombinant protein (1-146aa). The recombinant protein was mainly present in the inclusion bodies (lane 5 and 6). The purified HQT recombinant protein (purity up to 85%) was used to immunize rabbits.** Note: 1: 0.4mg/ml BSA; 2: Mark; 3-4: Supernatant; 5: Purified protein diluted 2x; 6: Purified protein diluted 10x.


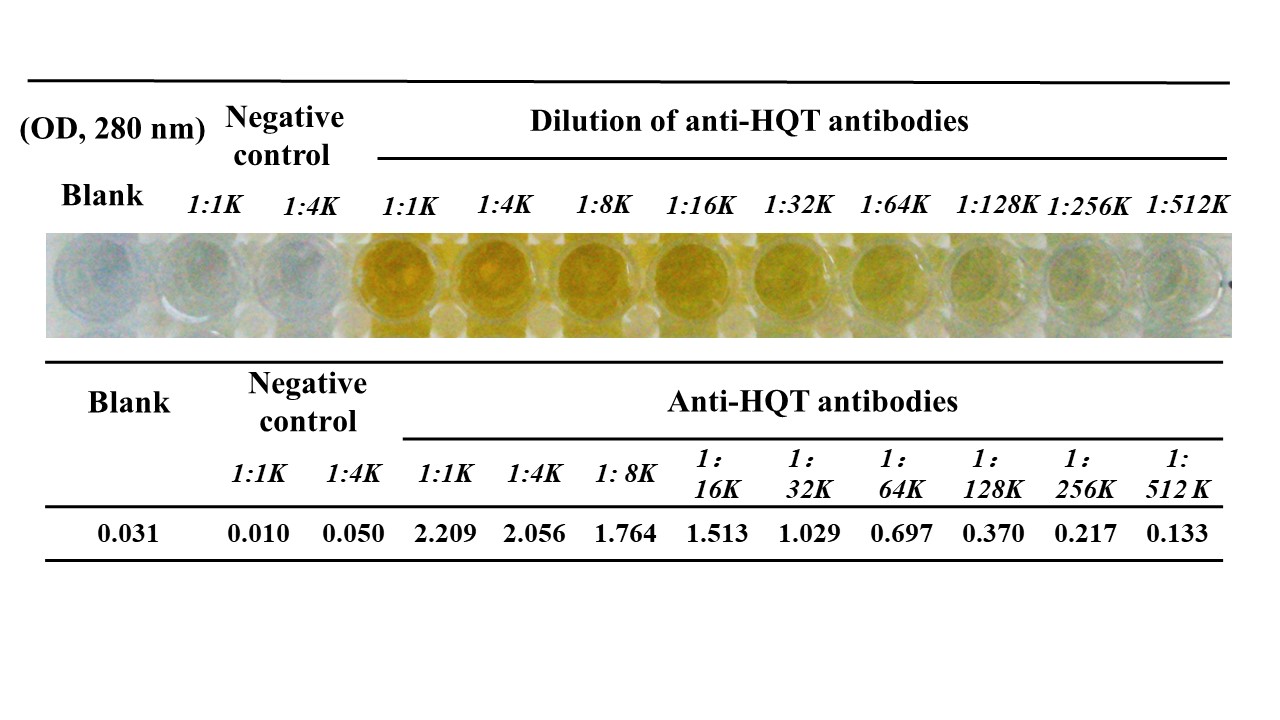


**Figure. S3. ELISA assay of the purified** **anti-HQT antibodies.**

Note: The ELISA assay (OD) at 280nm. The blank and the negative controls show very low activity when they were diluted at 1:1k or1:4k, but the anti-bodies show strong activities at various dilutions (up to 1:128k).


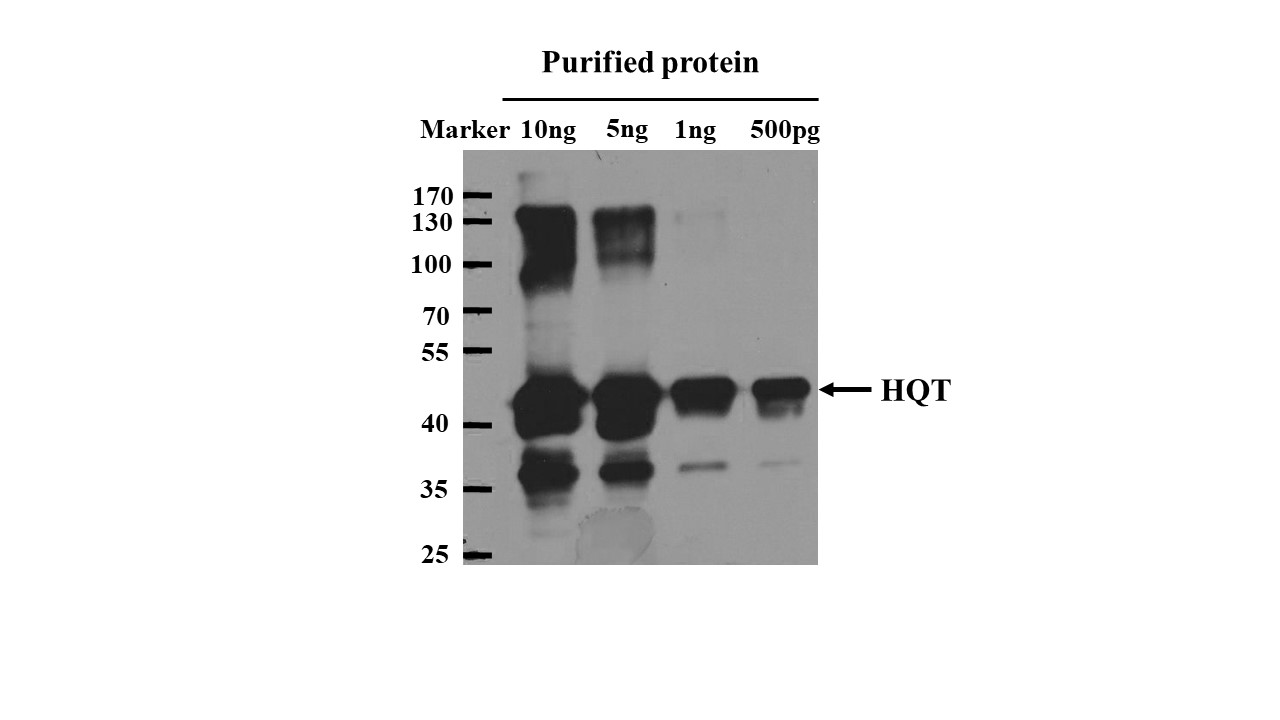


**Figure. S4. Western blot analysis of the purified anti-HQT antibodies (diluted 1:1000) against the** **purified recombinant HQT protein. The amounts of** **purified protein loaded were indicated on the top.**

**Table. S1**

**Prediction of the HQT locational signals using the TargetP software and ChloroP prediction tools**

<http://www.cbs.dtu.dk/services/TargetP/>, <http://www.cbs.dtu.dk/services/ChloroP/>

Amino acid sequence length of HQT is 439, cTP, mTP and SP were the scores for chloroplast protein, mitochondrial protein and secretory protein respectively. Loc is the position predicted by the website. RC is the credibility. cTP-length: estimated length of chloroplast signal peptide.

|  | Length | cTP | mTP | SP | other | Loc | RC | cTP-length |
| --- | --- | --- | --- | --- | --- | --- | --- | --- |
| HQT | 439 | 0.453 | 0.021 | 0.039 | 0.858 | C | 3 | 79 |

**References:**

Emanuelsson O., Nielsen N., Brunak S., Heijne G.V. ChloroP, a neural network-based method for predicting chloroplast transit peptides and their cleavage sites. Protein Science. 1999,8,978–984

Emanuelsson O, Nielsen H, Brunak S., Heijne G.V. Predicting Subcellular Localization of Proteins Based on their N-terminal Amino Acid Sequence. J. Mol. Biol. (2000) 300, 1005-1016.
